# Exploring the Functional Potential of the Broiler Gut Microbiome Using Shotgun Metagenomics

**DOI:** 10.3390/genes16080946

**Published:** 2025-08-11

**Authors:** Nuria Peña, Irene Lafuente, Ester Sevillano, Javier Feito, Gastón Allendez, Estefanía Muñoz-Atienza, Fiona Crispie, Luis M. Cintas, Paul D. Cotter, Pablo E. Hernández, Juan Borrero

**Affiliations:** 1 Departamento de Nutrición y Ciencia de los Alimentos (NUTRYCIAL), Sección Departamental de Nutrición y Ciencia de los Alimentos (SD-NUTRYCIAL), Facultad de Veterinaria, Universidad Complutense de Madrid (UCM), Avenida Puerta de Hierro, s/n, 28040 Madrid, Spain; nuriapen@ucm.es (N.P.); irelafue@ucm.es (I.L.); estsev01@ucm.es (E.S.); lcintas@vet.ucm.es (L.M.C.); ehernan@vet.ucm.es (P.E.H.); 2 Teagasc Food Research Centre, Moorepark, Co. Cork, APC Microbiome Ireland, P61 C996 Fermoy, Ireland

**Keywords:** bacteriocin, antimicrobial peptides, RiPPs, metagenome, poultry, AMR, virulence factor

## Abstract

**Background/Objectives**: Antimicrobial peptides (AMPs) have emerged as promising alternatives to conventional antibiotics in livestock, offering a sustainable strategy for controlling bacterial pathogens in food production systems. In addition to their direct antimicrobial effects, AMPs play a key role in modulating host-associated microbiomes, influencing both microbial composition and function. Advances in metagenomic sequencing and bioinformatic tools now enable comprehensive exploration of AMP diversity and activity within complex microbial ecosystems. **Methods**: In this study, we employed Illumina-based next-generation sequencing (NGS) to analyze intestinal contents from six gut sections of broiler chickens obtained from a Spanish slaughterhouse. **Results**: Through de novo assembly and bioinformatic annotation, we identified biosynthetic gene clusters (BGCs) encoding ribosomally synthesized and post-translationally modified peptides (RiPPs), other specialized bioactive secondary metabolites, antimicrobial resistance genes (ARGs), virulence factor genes (VFGs), and a diverse microbial community. Among all gut sections, the cecum exhibited the highest genetic richness, characterized by a high diversity of RiPP-like clusters and antimicrobial resistance determinants. **Conclusions**: These findings highlight the poultry gut, particularly the cecum, as a significant reservoir of antimicrobial peptides (AMPs) with potential implications in antibiotic-free poultry production and enhanced food safety.

## 1. Introduction

The growing threat of antimicrobial resistance (AMR) has become a global health crisis, as recognized by the World Health Organization [1]. One major contributor to this problem is the widespread use of antibiotics in livestock, especially in poultry farming, where antibiotics have been used to promote growth and prevent disease [2,3]. This practice has led to the emergence and spread of resistant pathogens, posing significant risks to both animal and human health through the food chain.

In accordance with the “One Health” framework, which underscores the interdependence of human, animal and environmental health, the European Union has implemented stricter regulations governing the use of antibiotics in animal agriculture. Notably, Regulation (EU) 2019/6 (Regulation (EU) 2019/6) [4] enforces stringent controls on antimicrobial administration in livestock to combat the emergence and spread of antimicrobial resistance. Concurrently, increasing public awareness of this global health threat has significantly influenced consumer preferences, driving demand for poultry meat and derived products marketed under labels such as ‘antibiotic-free’ (ABF) [5] “raised without antibiotics” (RWA) [6] or “no antibiotics ever” (NAE) [7].

Nevertheless, antibiotics remain a critical component of veterinary practice in poultry production, particularly for the treatment of infections and the prevention of disease outbreaks. This highlights the urgent need for safe, effective, and sustainable alternatives to conventional antimicrobials. Among the most promising strategies are the use of probiotics, prebiotics and symbiotic to beneficially modulate the gut microbiota [8,9] as well as the development of targeted vaccines to prevent specific bacterial and viral infections [10]. Other emerging approaches include bacteriophage therapy [11], phytogenic feed additives such as essential oils and plant extracts, competitive exclusion cultures [12] and antimicrobial peptides (AMPs) and bacteriocins [13,14,15], which offer promising antimicrobial activity with a lower risk of resistance development.

AMPs, particularly those synthesized by bacteria and known as bacteriocins, are receiving growing attention due to their well-defined spectrum of activity and reduced potential to induce resistance [16]. These ribosomally synthesized compounds exhibit diverse structures and mechanisms of action, and are typically encoded within well-organized BGCs, which facilitate both efficient production and self-immunity in the host organism. Traditionally, bacteriocins were categorized into class I, comprising peptides that undergo post-translational modifications, and class II, consisting of unmodified peptides [17]. However, current classification systems have expanded class I to include all ribosomally synthesized and post-translationally modified peptides (RiPPs), such as lanthipeptides, head-to-tail cyclized peptides, linear azol (in)e-containing peptides, thiopeptides, lasso peptides, sactipeptides, and others. Notably, five classes of lanthipeptides (I to V) have also been identified [18,19,20,21].

Importantly, bacteriocins are naturally produced within microbial communities, where they play a pivotal role in shaping community structure and dynamics by mediating inter-microbial competition and inhibiting the growth of pathogenic species [22]. The discovery and characterization of novel bacteriocin gene clusters hold substantial promise for addressing critical challenges in microbial infection control, human and animal health, food safety, and sustainable agriculture. To support these efforts, several specialized bioinformatic platforms such as BAGEL4 [23] BACTIBASE [24] and antiSMASH [25] have been developed to facilitate the identification and annotation of bacteriocins, RiPPs and other antimicrobial secondary metabolites. Despite these advances, much remains to be elucidated regarding the ecological functions, structural diversity, and environmental distribution of these compounds. Conventional methods such as in vitro screening and whole-genome sequencing (WGS) provide valuable insights but are often constrained by low throughput, limited scalability, and an inability to capture the full diversity of uncultivable microorganisms. In contrast, metagenomic approaches offer a powerful, high-resolution framework for analyzing complex microbial communities, enabling the identification of novel BGCs and unlocking previously inaccessible reservoirs of antimicrobial potential from diverse ecosystems [26,27,28].

The gastrointestinal tract (GIT) of poultry harbors a dense and taxonomically diverse microbial community that plays a central role in digestion, immune function, and overall animal health. In addition to these essential functions, the gut microbiota actively participates in microbial competition and defense through the production of bioactive compounds including bacteriocins, RiPPs, and other specialized metabolites with antimicrobial activity [29]. These compounds contribute to the maintenance of microbial homeostasis by inhibiting competing or pathogenic microorganisms, thereby supporting microbiome stability and host protection. Accordingly, the poultry gut represents a valuable and underexplored reservoir of naturally occurring antimicrobial agents with potential applications in animal health and food safety. In this study, we employed Illumina-based shotgun metagenomic sequencing to characterize the taxonomic composition, biosynthetic potential, and resistome across multiple intestinal sections of broiler chickens. Our findings underscore the functional richness of the cecal microbiota and highlight the power of metagenomic approaches to reveal hidden reservoirs of antimicrobial potential within the poultry GIT.

## 2. Materials and Methods

### 2.1. Sample Acquisition and Processing

**Figure 1:**
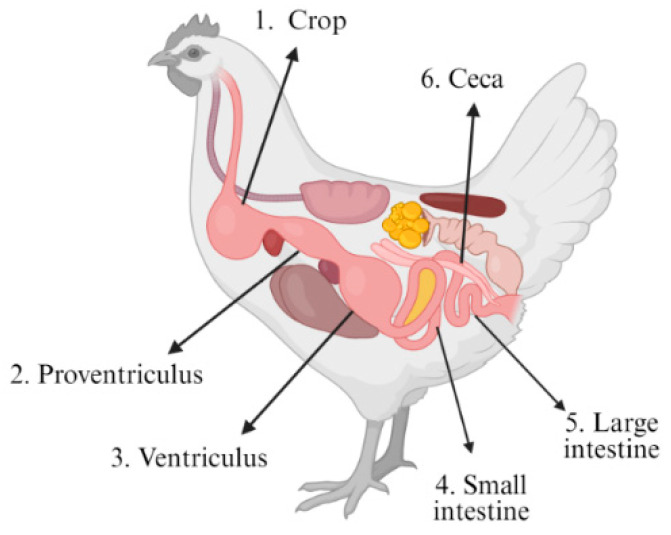
Schematic representation of the broiler gastrointestinal tract illustrating the six sampled sections: crop (CR), proventriculus (PR), ventriculus (VE), small intestine (SI), large intestine (LI), and ceca (CE). Figure created with BioRender.com (accessed on 31 November 2024).

Sample acquisition was carried out at a Spanish poultry slaughterhouse with an annual processing capacity exceeding 28,000 tons of chicken meat. However, due to the substantial economic costs associated with metagenomic sequencing for comprehensive genome profiling of gut microbiomes, this study focused on the analysis of six anatomically distinct sections of the GIT from broiler chickens. To this end, the GITs from two independently selected broiler chickens (designated as Broiler 1 [B1] and Broiler 2 [B2]) were collected immediately after the evisceration step on the processing line and transported on ice. The birds analyzed were commercial broilers of the Ross 308 strain, approximately 42 days old at the time of sampling. Upon arrival at the laboratory, each GIT was dissected into six defined anatomical sections: crop (CR), proventriculus (PR), ventriculus (VE), small intestine (SI), large intestine (LI), and ceca (CE) (Figure 1). From each section, 0.25 g of luminal content was aseptically collected for total DNA isolation.

### 2.2. DNA Extraction and Shotgun Metagenomic Sequencing

Total DNA was extracted from each sample using the QIAamp PowerFecal Pro DNA Kit (Qiagen, Hilden, Germany), following the manufacturer’s instructions. DNA concentration was measured using Qubit Fluorometer instrument Qubit Fluorometer (Thermo Fisher Scientific, Waltham, MA, USA). The integrity of the extracted DNA was evaluated by electrophoresis on a 1.5% (*w*/*v*) agarose gel (Pronadisa, Madrid, Spain), conducted at 90 V for 60 min. DNA bands were visualized using a GelDoc 1000 imaging system (Bio-Rad Laboratories, Hercules, CA, USA). Metagenomic libraries were prepared following the Illumina DNA Prep protocol and sequenced on the NovaSeq 6000 System (Illumina, San Diego, CA, USA) using the S4 Reagent Kit (300 cycles). Bioinformatics processing and data analysis were partially carried out by Microomics Systems S.L. (Barcelona, Spain).

### 2.3. Bioinformatics Analysis

#### 2.3.1. Metagenomic Read Processing, Assembly and Functional Annotation

Both forward and reverse Illumina reads underwent quality control using FastQC v0.11.8 [30] which generated descriptive quality metrics for each dataset. These results were subsequently aggregated and visualized using MultiQC v1.0 [31]. To assess potential contamination or off-target reads, paired-end sequences were screened against three reference genomes: the human genome (GRCh38), the *Gallus gallus* genome (GalGal1.mat.broiler. GRCg7b), and the *Sinsheimervirus* phiX174 genome (NC_001422), using FastqScreen v0.14.0 with Bowtie2 [32] as the aligner filtered (non-host) paired-end reads were then de novo assembled with MEGAHIT v1.2.9 [33] and assembly quality was evaluated with SeqKit v2.2.0 [34]. Contigs were annotated with Prokka v1.14.6 [35] using the metagenome flag to account for non-clonal sequences. For feature quantification, filtered reads were mapped back to the assembled contigs using Bowtie2 v2.3.5. Duplicate reads were identified and removed with Picard MarkDuplicates v2.27.1 [36] and gene-level feature counts were obtained using featureCounts v2.0.1 [37].

#### 2.3.2. Identification of Biosynthetic Gene Clusters and Virulence/Resistance Genes

BGCs involved in the production of specialized metabolites were identified using the antiSMASH v7.0 framework. This comprehensive tool enables the detailed annotation of predicted BGCs, including those responsible for the biosynthesis of RiPPs. Genome mining has emerged as a powerful approach for uncovering microbial metabolites. Through the analysis of genomic sequences, it is possible to identify secondary metabolite gene clusters (SMGCs) [38] including those encoding the biosynthesis of terpenes and terpenoids, which play important roles in signaling, defense mechanisms, host–microbe interactions, and stress responses [39]. Particularly noteworthy are gene clusters encoding polyketide synthases (PKSs) and non-ribosomal peptide synthetases (NRPSs), which are of particular interest due to their involvement in the production of clinically significant molecules with antimicrobial, antifungal, anticancer, antiviral, and other therapeutic properties [40].

Virulence-associated genes were identified by aligning sequences against the Virulence Factor Database (VFDB) [41], using minimum identity and coverage thresholds of 80%. ARGs were characterized using the Resistance Gene Identifier (RGI) v6.0.2 [42].

#### 2.3.3. KEGG-Based Functional Annotation

To evaluate the metabolic potential of the microbial communities, predicted coding sequences were functionally annotated against the Kyoto Encyclopedia of Genes and Genomes (KEGG) database [43]. Functional profiles were generated by mapping gene annotations to KEGG orthologs and pathways, enabling the identification of sequences involved in secondary metabolism, environmental adaptation, and other microbial functions.

#### 2.3.4. Taxonomy Profiling

The taxonomic profile was generated by classifying filtered (non-host) reads against a reference database comprising genomes and plasmids from Archaea, Bacteria, Viruses, Protozoa, and Fungi. Taxonomic classification was performed using Kraken2 v2.0.7 [44] and abundance estimates were subsequently refined with Bracken v2.5 [45].

## 3. Results

### 3.1. Assembly Quality Reports

From the 12 analyzed samples, a total of 250,440,052 raw sequences were generated. Following quality control and host-read removal procedures, 46,660,694 high-quality paired non-host reads were retained for downstream analysis.

Assembly results are summarized in Table 1. The number of paired non-host reads per sample ranged widely, ranging from 13,986 (B1_SI) to 24,098,100 (B1_CE), with a mean of approximately 511,690 reads (excluding the negative control). This variation was reflected in the number of contigs, which ranged from 3717 (B1_SI) to 500,066 (B1_CE). The average contig length across all samples was 2141 bp. Assembly contiguity, measured by N50 and L50, also exhibited substantial variability: N50 values ranged from 528 bp (B1_SI) to 3013 bp (B2_LI), while L50 values ranged from 1190 (B1_SI) to 50,213 (B1_CE). These metrics underscore the differences in sequencing depth, microbial composition, and sample complexity.

### 3.2. Identification of Biosynthetic Gene Clusters

**Figure 2:**
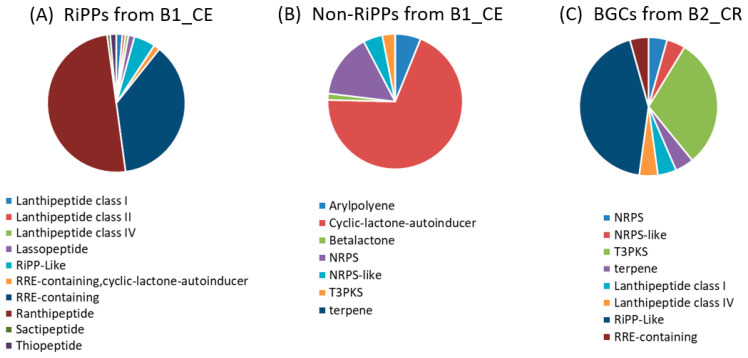
Distribution of BGCs predicted by antiSMASH in selected intestinal samples. (**A**) RiPP-related BGCs identified in sample B1_CE; (**B**) Non-RiPP BGCs detected in sample B1_CE; and (**C**) Total BGCs identified in sample B2_CR, including both RiPP and non-RiPP categories.

Using the antiSMASH v7.0 framework, putative BGCs associated with the production of specialized metabolites were identified. Sample B1_CE displayed the highest number of predicted BGCs with a total of 205 annotated regions (Figure 2A). Of these, 140 were classified as encoding RiPPs, positioning this class as the most prominent within the B1-CE microbiome. The majority of RiPP-associated BGCs were annotated as ranthipeptides and RiPP recognition element (RRE)-containing clusters. Additional RiPP subclasses included lanthipeptides, lasso peptides, sactipeptides, and thiopeptides a well-characterized group of RiPPs known for their potent and specific antimicrobial properties. The prevalence of RiPP-encoding BCGs, particularly those linked to antimicrobial activity, underscores the potential of the cecal microbiome as a rich reservoir of AMPs cecal microbiome and suggests a possible role in host-associated colonization resistance.

The remaining 65 BCGs identified in sample B1_CE were assigned to non-RiPP biosynthetic classes. Among these, the most frequently represented categories included type III polyketide synthases (T3PKS), non-ribosomal-peptide, synthetase-like (NRPS-like) clusters and β-lactone pathways (Figure 2B). These classes are widely recognized for their involvement in the biosynthesis of structurally diverse specialized secondary metabolites, many of which possess potent bioactivities, including antimicrobial, cytotoxic, and signaling functions.

While B1_CE emerged as the most biosynthetically enriched sample, the B2_CR sample also exhibited a moderate repertoire of BGCs, with a total of 23 predicted regions, some classified as RiPP-related and others as non-RiPP (Figure 2C). In contrast, the remaining intestinal samples contained only a few detectable BGCs, reinforcing the idea that the biosynthesis of secondary metabolites is highly compartmentalized within the gastrointestinal tract and likely shaped by both anatomical location and host-specific microbiota composition.

### 3.3. Antimicrobial Resistance Genes Profile

**Figure 3:**
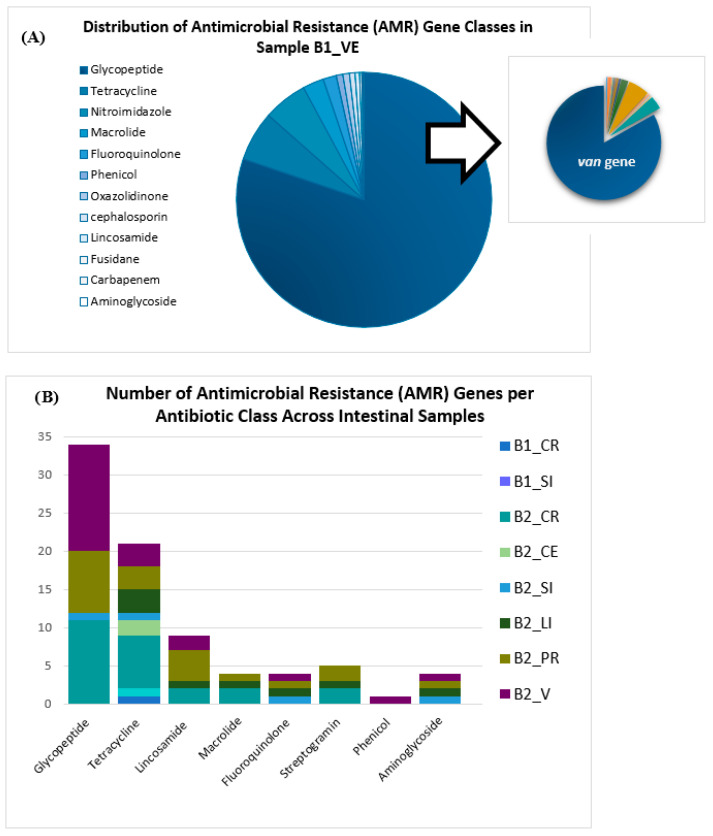
ARGs profiles identified in broiler intestinal samples. (**A**) Distribution of ARGs classes detected in sample B1_VE, categorized by antibiotic class. Each color in the pie chart represents a different antibiotic class. Glycopeptide resistance genes were predominant, with the inset highlighting the dominance of the *van* gene family (dark blue) over other gene types (other colors). (**B**) Stacked bar chart illustrating the number of ARGs per antibiotic class across all analyzed intestinal samples. Colors in the bars correspond to different samples, as indicated in the legend.

ARGs were identified across the intestinal samples, with sample B1_VE displaying the most extensive resistance profile. A total of 712 ARGs were identified in this sample, of which 571 (80.2%) were associated with glycopeptide resistance. Notably, 83.2% of these glycopeptide-related ARGs were classified as variants of the van gene family. In addition, the resistome of B1_VE included 45 genes conferring resistance to tetracyclines (6.3%), along with single detections of genes conferring resistance to carbapenems and aminoglycosides (0.14% each). The relative abundance of resistance gene categories in B1_VE is shown in Figure 3A.

In the remaining samples, glycopeptide resistance genes remained the most frequently detected ARG category, accounting for 41.5% of the total resistance genes identified. This was followed by tetracycline resistance genes (25.6%) and lincosamide resistance genes (10.1%). Other resistance classes, including those conferring resistance to streptogramins, phenicols, and macrolides, were detected at lower frequencies, generally comprising less than 5% of the total ARGs (Figure 3B). Among these samples, B2_CR, B2_VE, and B2_PR exhibited the highest ARG burdens, with 24 (29.3%), 22 (26.8%), and 20 (24.4%) resistance-related genes, respectively. These data underscore notable sample-specific variability in both the abundance and composition of ARG profiles, with sample B1_VE standing out due to its unusually high enrichment in resistance determinants.

### 3.4. Identification of Virulence Factor Genes (VFGs)

**Figure 4:**
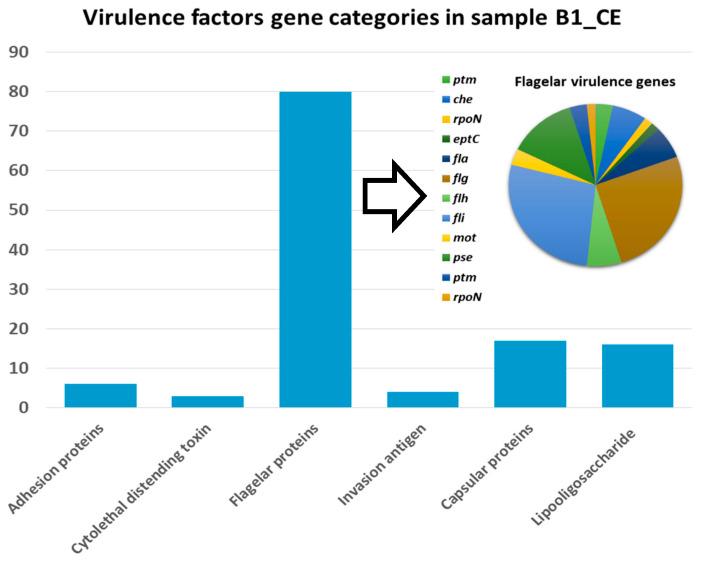
Virulence factor gene profile in sample B1_CE. The bar chart illustrates the number of genes associated with key virulence factor categories, including flagellar components, capsular proteins, lipo-oligosaccharides biosynthesis enzymes, and adhesion-related factors. The inset pie chart provides a detailed breakdown of flagellar-associated genes.

Virulence factor genes (VFGs) were predominantly detected in sample B1_CE, which accounted for over 97% of all virulence-associated annotations across the dataset (Figure 4). The majority of these genes were linked to bacterial motility and surface-associated structures, with a substantial proportion corresponding to flagellar components, particularly genes belonging to the encoded *fli* and *flg* gene families, which together comprised nearly two-thirds of the total VFGs identified. This marked enrichment likely reflects the competitive and metabolically active environment of the cecum, where motility confers ecological advantages such as chemotaxis, colonization, and spatial niche adaptation. Beyond their role in locomotion, flagella also contribute to processes including adhesion, biofilm formation, protein secretion, and host-pathogen interactions underscoring their multifaceted functional relevance. Other VFGs were implicated in key pathogenicity-related functions, including capsule biosynthesis, lipo-oligosaccharide production, and bacterial adhesion to host tissues (Figure 4).

### 3.5. Functional Annotation of Metabolomic Compounds

Metabolic functional profiling of all samples was conducted by annotating coding sequences against the Kyoto Encyclopedia of Genes and Genomes (KEGG) database. The number and diversity of pathway-associated sequences varied substantially among samples, with the highest metabolic potential observed in the cecal samples from both broilers.

**Figure 5:**
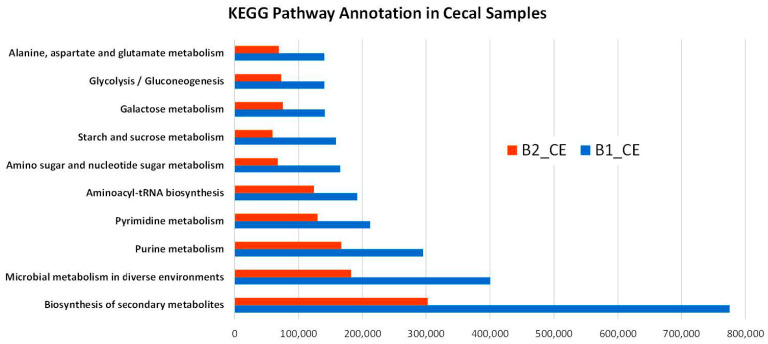
Functional classification of KEGG-annotated sequences in the cecal samples of broilers. The bar chart shows the total number of coding sequences assigned to major KEGG functional categories in the cecal samples B1_CE and B2_CE.

Sample B1_CE showed the highest number of KEGG-annotated sequences (2,619,603 hits), followed by B2_CE (1,250,357 hits). In both cases, the most represented functional categories were “Biosynthesis of secondary metabolites” (KEGG pathway 01110) and “Microbial metabolism in diverse environments” (KEGG pathway 01120), together accounting for over 44% of all annotations in each sample. This enrichment suggests a high level of biosynthetic and ecological activity within the cecal microbiota. The comparative distribution of pathway annotations between B1_CE and B2_CE is shown in Figure 5, while the complete KEGG annotation counts for all samples are provided in Supplementary Table S1. Together, these results highlight the functional enrichment of cecal microbiota, particularly in metabolic processes linked to environmental adaptation and secondary metabolite production.

### 3.6. Microbial Taxonomic Profiling of Intestinal Microbiomes Across Sample Sites

**Figure 6:**
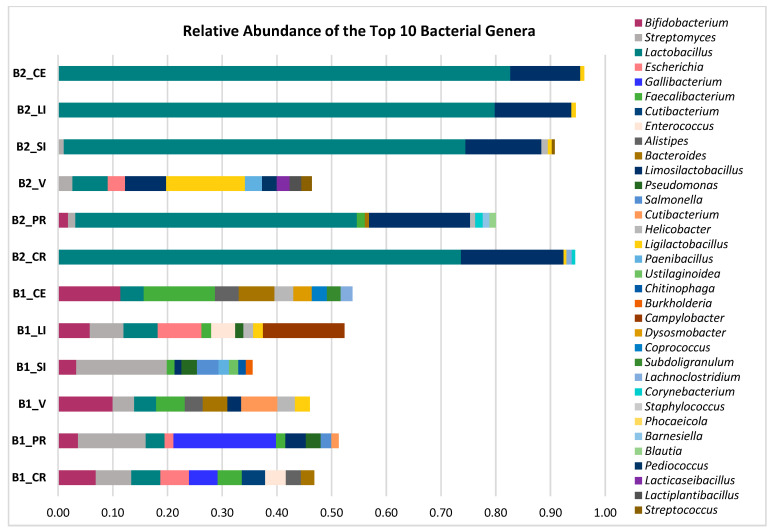
Genus-level taxonomic composition across intestinal samples from broilers. Bar plots represent the relative abundance of the top 10 most prevalent bacterial genera identified in each sample.

Taxonomic profiling was performed on all intestinal samples to evaluate the composition and relative abundance of microbial taxa across distinct anatomical regions. Based on Illumina sequencing data and de novo assembly using MEGAHIT, the analysis revealed considerable variability in microbial diversity and community structure among the 12 intestinal samples. Notably, genus-level composition exhibited marked differences across sites. Focusing on the top 10 most abundant genera per sample, a total of 34 unique genera were identified (Figure 6), underscoring both the richness and spatial heterogeneity of the intestinal microbiota. These findings highlight the influence of gut compartmentalization and host-specific factors on microbial community assembly.

Across all intestinal samples, *Lactobacillus* emerged as the most dominant genus, representing 49.6% of the total reads and detected in every sample except B1_SI. Other genera with notable prevalence included *Limosilactobacillus* (11.8%), *Streptomyces* (6.5%), and *Bifidobacterium* (5.5%). In contrast, genera such as *Chitinophaga*, *Burkholderia*, *Staphylococcus*, *Phocaeicola*, *Barnesiella*, and *Blautia* were present at very low relative abundances, ranging from 0.012% to 0.15%, indicating their limited contribution to the overall community structure.

Samples exhibited distinct microbial diversity patterns. Four samples displayed highly skewed community structures, in which the top 10 genera accounted for nearly the entirety of detected sequences (relative abundance ≈ 1), indicating low taxonomic evenness. In contrast, the majority of samples showed more balanced microbial profiles, with the top 10 genera comprising less than 60% of the total relative abundance, suggesting greater microbial diversity and community complexity.

**Figure 7:**
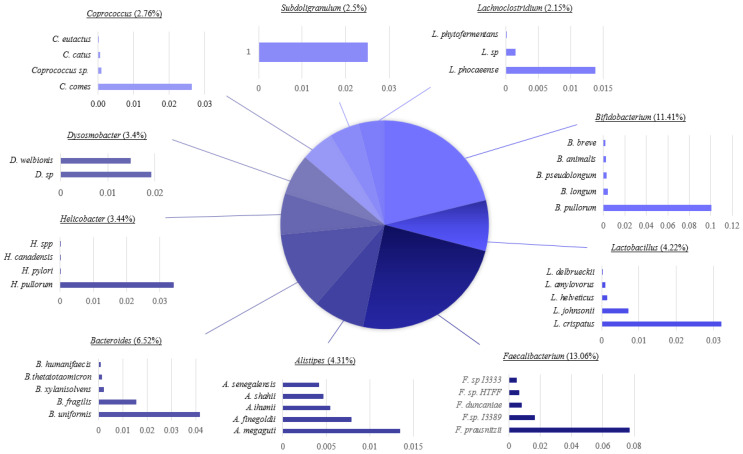
Taxonomic composition of cecal sample B1_CE at the genus level. The pie chart displays the relative abundance of the 10 most prevalent bacterial genera identified in the sample.

More detailed taxonomic analyses were conducted for samples B1_CE and B2_CE. Figure 7 displays the genus-level taxonomic composition of B1_CE, which exhibited a relatively balanced microbial community. The most dominant genera included *Faecalibacterium* (13%), *Bifidobacterium* (11%), and *Bacteroides* (6.2%). At species-level resolution, the sample revealed a diverse array of prevalent taxa, notably *Faecalibacterium prausnitzii*, *Bifidobacterium pullorum*, and *Lactobacillus crispatus*. In contrast, Supplementary Figure S1 illustrates the taxonomic profile of B2_CE, which was heavily dominated by *Lactobacillus* (82.6%) and *Limosilactobacillus* (12.7%). Even within these dominant genera, species-level composition was less evenly distributed compared to B1_CE, with *L. crispatus*, *L. reuteri*, and *L. salivarius* comprising the majority of reads. Notably, *L. crispatus* emerged as one of the most abundant species in both cecal samples, suggesting it may represent a core component of the broiler cecal microbiome.

## 4. Discussion

Global poultry meat production has grown rapidly since the 1960s, driven largely by the cost-efficiency of intensive farming systems [46]. However, this growth has brought ongoing challenges that require continuous reassessment of production practices. One of the most pressing concerns is the widespread use of antibiotics for disease prevention and growth promotion. This practice has raised alarm due to its role in the emergence of AMR. In parallel, contamination of poultry products with foodborne pathogens remains a persistent risk throughout the production chain [47,48]. In response, the poultry industry is increasingly transitioning toward “antibiotic-free” production models. Achieving this goal requires identifying alternative antimicrobial strategies, including the identification, evaluation and use of novel bioactive compounds and the strain producers. In this context, AMPs have emerged as promising candidates due to their broad-spectrum activity and lower propensity for inducing resistance compared to conventional antibiotics.

The GIT harbors a diverse and dynamic microbial community capable of producing a broad array of bioactive metabolites, including compounds with potential antibacterial activity. Notably, bacteriocins and RiPPs, among other classes, have emerged alongside other bioactive compounds as promising antimicrobial candidates to supplement or replace conventional antibiotics in poultry production. Advances in high-throughput sequencing technologies have transformed microbiome research by enabling comprehensive analysis of unculturable microorganisms and their functional potential across diverse environments including food systems, soil, and host-associated microbiomes [49,50,51]. Despite these technological developments, the recovery of high-quality metagenomic data from complex microbial communities remains a significant challenge. In the present study, we employed Illumina-based shotgun metagenomic sequencing, coupled with established bioinformatics pipelines, to investigate the biosynthetic potential and taxonomic composition of microbial communities across multiple anatomical sections of the GIT of broiler chickens.

Sequencing and assembly quality varied markedly across the GIT samples analyzed, with many exhibiting lower-than-expected read counts and suboptimal assembly metrics particularly in terms of contig number and length. Notably, the cecal samples, and especially sample B1_CE, stood out for their superior sequencing deep, longer average contig lengths, and more favorable assembly statistics, including higher N50 and lower L50 values. In contrast, assemblies from most other anatomical sites were more fragmented, with shorter contigs that limited the effective recovery of metagenomic features (Table 1). These disparities are likely attributable, at least in part, to biological differences, as the cecum is recognized as a densely populated and taxonomically diverse microbial niche. It plays key physiological roles in fermentation, nutrient absorption, polysaccharide degradation, water reabsorption and nitrogen recycling [52,53,54,55,56], which may contribute to enhanced microbial representation and sequencing performance in this region. Other gut sections with lower microbial density or less stable communities may pose challenges for comprehensive metagenomic reconstruction, emphasizing the importance of sample selection and anatomical context in microbiome studies. Technical variables such as DNA extraction efficiency and sample integrity, may have also contributed to the lower assembly quality observed in distinct gut compartments.

Notably sample B1_CE exhibited the highest number of BGCs, with a particular enrichment of those associated with AMPs (Figure 2). The antiSMASH analysis identified sequences across distinct categories, with RiPP-like clusters being the most prevalent [57,58,59]. Although many predicted RiPPs in this sample could not be confidently assigned to specific subtypes, a subset was annotated as lanthipeptides (types I and IV), lasso peptides, thiopeptides, and sactipeptides. This classification may reflect limitations in read length or assembly quality, which can hinder complete gene cluster reconstruction. Additional investigation into these RiPP-related sequences through targeted assembly refinement or long-read sequencing, could provide greater insight into their structural diversity and potential antimicrobial activity, particularly given their established roles in microbial competition and quorum sensing [60]. In addition, the synthesis, bioactivity and ecological functions of these predicted clusters warrant further investigation to determine their specific roles within the gut microbiome.

ARG profiling revealed marked differences among the samples, with sample B1_VE displaying an exceptionally high ARG burden (Figure 3). This sample alone contained a high number of ARGs which were associated with glycopeptide resistance. Most of these genes belonged to the van gene family, particularly *vanA*, which is known to confer high-level resistance to vancomycin and teicoplanin and is frequently associated with mobile genetic elements such as transposon Tn1546 [61,62]. This observation is consistent with previous reports connecting glycopeptide resistance to *Enterococcus faecium*, *E. faecalis*, and *Enterococcus hirae*, as well as commensal genera such as *Lactobacillus* and *Lactococcus* [63,64,65,66,67]. Notably, several of these genera were among the most abundant taxa identified across the samples in this study, suggesting that dominant community members may play a role in the dissemination and persistence of ARGs within the poultry gut ecosystem. In addition, the observed heterogeneity in ARG distribution may reflect underlying differences in microbial community structure, anatomical niche characteristics, or previous exposure to antimicrobial selective pressures.

Tetracycline resistance genes were the second most abundant class of ARGs. These antibiotics are known to be poorly metabolized by animals, which increases selective pressure on gut microbiota [68]. Other resistance categories including lincosamides, macrolides and phenicol were detected at lower frequencies. Overall, the resistome composition varied across samples and individuals, with B1_VE exhibiting the highest level of enrichment. However, due to the limited number of samples analyzed and the inherent variability of metagenomic data across gut compartments and hosts, these findings should be interpreted with caution. Larger-scale studies are needed to determine whether the patterns observed here represent broader trends in poultry resistomes or are specific to the individuals included in this study.

The cecal sample B1_CE stood out also for harboring the greatest number of predicted VFG, which were almost exclusively detected in this sample (Figure 4). The majority of these genes were associated with flagellar proteins, suggesting a prominent role for motility-related functions in microbial competitiveness within this gut region [69]. Beyond facilitating movement, flagella contribute to key processes such as bacterial adhesion, chemotaxis, colonization, and biofilm formation, traits known to enhance survival and persistence in densely populated microbial ecosystems [70,71,72]. In addition, the presence of additional VFGs involved in capsule biosynthesis, lipo-oligosaccharide production, and adhesion further suggests that specific constituents of the cecal microbiota may harbor virulence-associated traits with potential implications for host interaction and microbial competitiveness.

Metabolic functional profiling of all samples was performed by annotating coding sequences against the KEGG database. Although the number and diversity of pathway-associated sequences varied markedly among samples, the highest metabolic potential was observed in the cecal samples B1_C and B2_CE from the broilers (Figure 5). These results highlight a functional enrichment of the cecal microbiota, particularly in metabolic pathways related to environmental adaptation and secondary metabolite biosynthesis. Overall, these findings emphasize the functional specialization of the cecal microbiome, particularly in metabolic pathways associated with environmental adaptation and secondary metabolite biosynthesis.

Taxonomic profiling was performed on all intestinal samples to evaluate the composition and relative abundance of microbial taxa across distinct anatomical regions (Figure 6). Marked differences in microbial composition and structure were observed among regions. Samples from the crop and cecum (e.g., B2_CR and B1_CE) exhibited higher taxonomic diversity, characterized by a more even distribution of genera such as *Limosilactobacillus*, *Bifidobacterium*, and *Streptomyces.* In contrast, samples from the proximal small intestine (e.g., B1_SI and B2_SI) demonstrated reduced diversity and were often dominated by a single genus, such as *Lactobacillus*. The dominance of *Lactobacillus* aligns with prior studies identifying it as major and functionally relevant constituent of the broiler cecal microbiota [73]. *Lactobacillus* species are well known for producing bacteriocins and other antimicrobial metabolites that exhibit inhibitory activity against key poultry pathogens, including *Staphylococcus aureus*, *Escherichia coli*, and *Salmonella typhi* [74]. *L. crispatus* was notably enriched in sample B1_CE, a species frequently associated with enhanced host health and microbiota stability in poultry [75,76].

The cecal samples (B1_CE and B2_CE), as shown in Figure 7 and Supplementary Figure S1, displayed a microbiota that was both functionally and taxonomically enriched, consistent with the cecum’s role in fermentation and microbial metabolism. This was supported by the high abundance of genera like *Streptomyces* and *Bacteroides*, linked to secondary metabolite production and fiber degradation. These findings suggest that microbial composition is shaped by anatomical location and host-specific factors such as oxygen levels, pH, substrate availability, and microbial competition. Collectively, these results underscore both the broad taxonomic diversity and the host-specific microbial profiles, highlighting potential ecological divergence driven by host physiology, anatomical site, or environmental exposure.

This study highlights the potential of shotgun metagenomics to uncover the biosynthetic diversity and antimicrobial capacity of the poultry GIT, particularly the cecum. While larger-scale metagenomic studies have typically assessed the poultry gut microbiome as a whole [77,78,79,80], few have resolved biosynthetic and functional features across anatomically distinct intestinal regions. Despite the limited number of samples analyzed, this study offers valuable exploratory insights into the biosynthetic potential, antimicrobial resistance, and taxonomic diversity of the broiler gut microbiome. The scarcity of metagenomic studies focused on poultry intestinal sections underscores the relevance of our findings. This study was designed as an exploratory proof-of-concept, with the primary aim of evaluating the feasibility of using shotgun metagenomics to characterize the biosynthetic, resistome, and taxonomic features of the broiler gut microbiome along different intestinal regions, rather than to produce statistically representative or population-wide conclusions. By characterizing the microbiota across multiple gut compartments, we provide a unique perspective on the spatial distribution of BGCs, ARGs, and virulence-associated traits. Notably, the cecum emerged as a metabolically active and taxonomically diverse microbial niche, enriched in BGCs encoding RiPPs and other bioactive secondary metabolites, as well as ARGs and VFGs. Future studies should build upon these results by incorporating larger animal cohorts and prioritizing the experimental validation of BGCs, alongside comprehensive functional analyses of their encoded antimicrobial peptides. Such efforts will be essential to elucidate their ecological significance and therapeutic potential within the gut microbiome.

The integration of synthetic biology tools, such as gene cloning, heterologous expression systems and in vitro cell-free protein synthesis (IV-CFPS) [81,82] offers powerful strategies for the functional characterization and scalable production of these candidate AMPs. Such approaches will be instrumental in evaluating their antimicrobial efficacy, spectrum of activity, and potential ecological roles within the gut microbiome, thereby advancing their translational implication in sustainable animal agriculture and antimicrobial stewardship. Future studies should also include host-level analyses, such as transcriptomic, immunological, or histopathological profiling, to better understand the physiological relevance and potential impact of microbiome-derived AMPs on gut health and colonization resistance. In addition, future studies should consider the use of long-read sequencing technologies, such as PacBio or Oxford Nanopore, to improve assembly quality and enable the complete reconstruction of biosynthetic gene clusters and associated mobile genetic elements. Longitudinal designs involving multiple flocks, breeds, and production systems would further enhance the robustness and generalizability of the findings.

## Figures and Tables

**Table 1:** Assembly metrics for each sample based on retained non-host reads. Summary of key assembly quality indicators across samples, including the number of paired non-host reads used for assembly, total contig count, average contig length, and assembly continuity statistics (N50 and L50).

		Paired_Non.Host_Reads	Contigs	Avg_Len (pb)	N50	L50
Broiler 1	CR	413,410	10,846	623	680	2758
	PR	221,628	6881	576	611	2779
	VE	417,875	12,591	5996	631	3560
	SI	13,986	3717	515	528	1190
	LI	405,471	9913	6293	691	2371
	CE	24,098,100	500,066	9687	1516	50,213
Broiler 2	CR	5796,495	46,447	10,205	2170	3167
	PR	374,194	11,295	6167	663	2950
	VE	1,014,566	18,603	705	734	4043
	SI	657,987	19,464	7914	937	3579
	LI	3,519,182	32,411	10,936	3013	1693
	CE	9,727,800	52,119	10,353	2788	2960
	Avg	511,689.75	10,253	2141.16	1246.833	6771.91

## Supplementary Materials

The following supporting information can be downloaded at: https://www.mdpi.com/article/10.3390/genes16080946/s1, Table S1: Total number of coding sequences annotated to KEGG functional pathways across intestinal samples; Figure S1: Taxonomic composition of cecal sample B2_CE at the genus level.
