# Supplementary material for: Exploring the Functional Potential of the Broiler Gut Microbiome Using Shotgun Metagenomics
Source: Genes (Basel). 2025 Aug 11;16(8):946. doi: 10.3390/genes16080946 (PMC12385557; doi:10.3390/genes16080946)
Supplement: Supplementary file 1 [file genes-16-00946-s001.zip › genes-3787197-supplementary.pdf]

| KEGG Pathway Code | B1_CR | B1_PR | B1_VE | B1_SI | B1_LI | B1_CE | B2_CR | B2_PR | B2_VE | B2_SI | B2_LI | B2_CE |
|---|---|---|---|---|---|---|---|---|---|---|---|---|
| 01110 | 91 | 72 | 262 | 11 | 80 | 775,437 | 162,458 | 923 | 11,844 | 6,788 | 10,5908 | 302,688 |
| 01120 | 87 | 45 | 176 | 9 | 55 | 400,157 | 101,506 | 590 | 6,699 | 4,081 | 64,664 | 182,211 |
| 00230 | 31 | 22 | 106 | 0 | 17 | 295,368 | 92,230 | 503 | 9,158 | 3,733 | 59,855 | 166,781 |
| 00240 | 22 | 18 | 61 | 4 | 17 | 211,992 | 73,475 | 394 | 6,830 | 2,772 | 48,397 | 129,619 |
| 00970 | 0 | 0 | 80 | 0 | 0 | 191,584 | 68,218 | 342 | 4,548 | 2,539 | 43,641 | 124,178 |
| 00520 | 0 | 0 | 58 | 0 | 3 | 165,119 | 35,179 | 214 | 3,523 | 1,611 | 24,480 | 67,557 |
| 00500 | 11 | 7 | 18 | 2 | 7 | 158,220 | 30,169 | 181 | 1,742 | 1,415 | 20,782 | 59,483 |
| 00052 | 0 | 0 | 52 | 0 | 0 | 140,983 | 37,615 | 215 | 2,967 | 1,665 | 24,935 | 75,540 |
| 00010 | 18 | 16 | 85 | 9 | 21 | 140,429 | 37,660 | 247 | 3,921 | 1,901 | 25,976 | 72,922 |
| 00250 | 0 | 0 | 25 | 0 | 0 | 140,314 | 39,476 | 198 | 2,627 | 1,748 | 26,222 | 69,378 |
| Total | 260 | 180 | 923 | 35 | 200 | 2,619,603 | 677,986 | 3,807 | 53,859 | 28,253 | 444,860 | 1,250,357 |

01110: Biosynthesis of secondary metabolites
01120: Microbial metabolism in diverse environments
00230: Purine metabolism
00240: Pyrimidine metabolism
00970: Aminoacyl-tRNA biosynthesis
00520: Amino sugar and nucleotide sugar metabolism
00500: Starch and sucrose metabolism
00052: Galactose metabolism
00010: Glycolysis / Gluconeogenesis
00250: Alanine, aspartate and glutamate metabolism

**Supplementary Table S1.** Total number of coding sequences annotated to KEGG functional pathways across intestinal samples. Rows correspond to specific KEGG categories (identified by code and name), while columns represent samples from broilers. The heatmap color scale ranges from purple, indicating higher sequence counts, to green, indicating lower counts for each functional category.

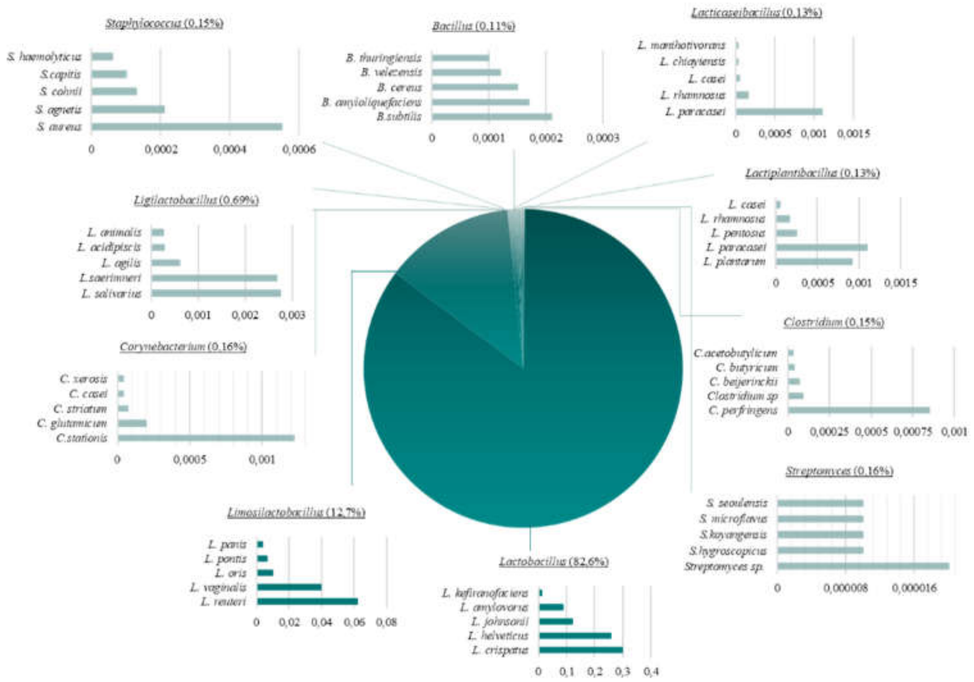


**Supplementary Figure S1.** Taxonomic composition of cecal sample B2_CE at the genus level. The pie chart shows the relative abundance of the 10 most prevalent bacterial genera identified in the sample.
